# Membrane vesicles of *Lacticaseibacillus rhamnosus* JB-1 contain immunomodulatory lipoteichoic acid and are endocytosed by intestinal epithelial cells

**DOI:** 10.1038/s41598-021-93311-8

**Published:** 2021-07-02

**Authors:** Kevin Champagne-Jorgensen, M. Firoz Mian, Karen-Anne McVey Neufeld, Andrew M. Stanisz, John Bienenstock

**Affiliations:** 1 Neuroscience Graduate Program, McMaster University, Hamilton, ON Canada; 2 Brain-Body Institute, St. Joseph’s Healthcare Hamilton, Hamilton, ON Canada; 3 Department of Pathology and Molecular Medicine, McMaster University, Hamilton, ON Canada; 4 McMaster Brain-Body Institute, St. Joseph’s Healthcare Hamilton, Juravinski Tower Room T3330, 50 Charlton Ave East, Hamilton, ON L8N 4A6 Canada

**Keywords:** Immunology, Microbiology

## Abstract

Intestinal bacteria have diverse and complex influence on their host. Evidence is accumulating that this may be mediated in part by bacterial extracellular membrane vesicles (MV), nanometer-sized particles important for intercellular communication. Little is known about the composition of MV from gram-positive beneficial bacteria nor how they interact with intestinal epithelial cells (IEC). Here we demonstrate that MV from *Lacticaseibacillus rhamnosus* JB-1 are endocytosed in a likely clathrin-dependent manner by both mouse and human IEC in vitro and by mouse IEC in vivo. We further show that JB-1 MV contain lipoteichoic acid (LTA) that activates Toll-like receptor 2 (TLR2) and induces immunoregulatory interleukin-10 expression by dendritic cells in an internalization-dependent manner. By contrast, neither LTA nor TLR2 appear to be required for JB-1 MV endocytosis by IEC. These results demonstrate a novel mechanism by which bacterial MV can influence host physiology and suggest one potential route for beneficial influence of certain bacteria and probiotics.

## Introduction

Intestinal microbes (microbiota) have complex bidirectional relationships with their host. In mammals, gut bacteria profoundly influence intestinal homeostasis, metabolism, and immunity, both developmentally^1^ and in adults^2^. As evidence for their diverse and often beneficial influence has increased, so too has interest in developing probiotics, bacterial supplements to improve intestinal health, treat disease, and even modulate mood^3,4^. Various mechanisms by which gut bacteria may influence their host have been proposed, including interactions between conserved bacterial components and host receptors, and secretion of bacterially-produced neurotransmitters, short-chain fatty acids, and other products^2,5,6^. However, despite strong commercial and academic scientific research interest, studies of these candidates have had limited success in fully uncovering the pathways involved.

One potential communication system, currently understudied in this context, involves extracellular vesicles, membrane-bound nanoparticles that are ubiquitously produced by both prokaryotes and eukaryotes^7^. In bacteria, these are often referred to as microvesicles or membrane vesicles (MV), or more specifically outer membrane vesicles (OMV) in the case of gram-negative bacteria. While OMV have been recognized for decades, it is only recently that release of MV through the thick gram-positive peptidoglycan cell wall was considered plausible^8^. As a result, while OMV have been well characterized for their roles in bacterial survival, pathogenesis, and immune modulation^9^, relatively little is known about the physiological relevance of gram-positive MV. In general, bacterial MV are known to contain active cargo reflecting the parent bacterium including toxins, virulence factors, nucleic acids, and other components^10^, which play an important role in interbacterial^11^ and bacteria-host communication^12^, and indeed may directly influence host gene transcription^13^.

A role for bacterial MV in activity related to beneficial bacteria was demonstrated by Shen et al.^14^, who found that OMV from the gram-negative commensal *Bacteroides fragilis* had immunoregulatory effects similar to the parent bacterium, and that these were mediated by OMV-associated polysaccharide A. Similarly, we have previously shown that the gram-positive bacterium *Lacticaseibacillus rhamnosus* JB-1 (JB-1; recently reclassified from *Lactobacillus*^15^ and previously misclassified as *L. reuteri*^16^) and its MV can promote the number and functions of regulatory T cells, activate TLR2, and induce an immunoregulatory phenotype in dendritic cells (DCs)^17–19^. Substantial evidence now exists that MV from beneficial bacteria can independently influence the host^20^, though their mechanisms of action in many cases remain unclear.

Bacteria express conserved features (microbe-associated molecular patterns; MAMPs) that are recognized by pattern recognition receptors (PRRs) of many host cells, including epithelial, endothelial, and immune^21^. These MAMPs are thought to be important signalling molecules also present on MV, which can modulate responses in host intestinal epithelial cells (IEC) and immune cells^9^ and may be involved in their immunoregulatory influence. Indeed, interactions between PRRs and MAMPs from commensal microbes are necessary to maintain homeostasis between host and microbiota, and thus contribute to healthy host development and immune responses^22^. Whether MAMPs are involved in activity associated with gram-positive MV is unknown.

Since we remain uncertain as to the exact mechanisms whereby gut bacteria influence the host, we sought to extend our previous work with JB-1 MV to learn more about their interaction with the gut epithelium and to further characterize their influence on dendritic cells. Here, we show that MV from JB-1 are endocytosed in a likely clathrin-mediated manner by both mouse and human IEC lines in vitro and by mouse IEC in vivo, using OMV from *Escherichia coli* Nissle 1917 (EcN) as a positive control as they are known to be endocytosed by IEC^23^. We further demonstrate that JB-1 MV contain lipoteichoic acid (LTA), which is recognized by TLR2 and induces interleukin 10 (IL-10) expression in DCs in vitro. Moreover, our data suggest that internalization of JB-1 MV by DCs is involved in their induction of IL-10 expression.

## Results

### Physical characterization of bacterial membrane vesicles

**Figure 1:**
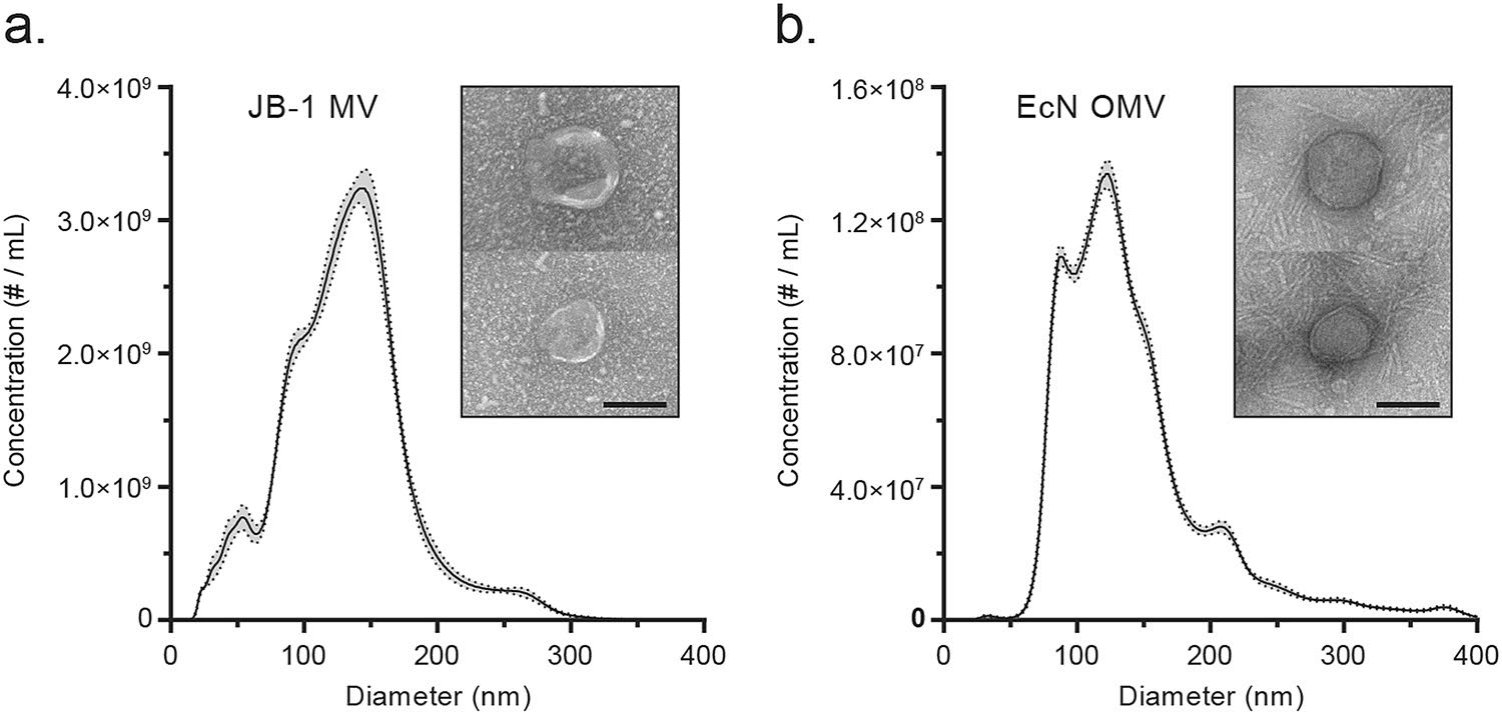
Characterization of bacterial MV. Membrane vesicles from (**a**) *L. rhamnosus* JB-1 and (**b**) OMV from *E. coli* Nissle 1917 were enumerated by nanoparticle tracking analysis (graphs) and visualized by transmission electron microscopy (inset images). Scale bars represent 100 nm. Ribbon represents ± 1 standard error of 5 technical replicates.

Membrane vesicles from *L. rhamnosus* JB-1 (JB-1) and *E. coli* Nissle 1917 (EcN) were collected by ultracentrifugation of cell-free culture broth and characterized by nanoparticle tracking analysis, transmission electron microscopy, and protein content. JB-1 MV preps had approximately 200 μg/mL protein and 3.1 × 10^11^ particles/mL, with a mean size of 130 nm (mode: 145 nm) (Fig. 1a). By contrast, EcN OMV had approximately 100 μg/mL protein and 1.2 × 10^10^ particles/mL, with a mean size of 145 nm (mode: 120 nm) (Fig. 1b). We found little variability in these characteristics between independent preparations.

### *L. rhamnosus* JB-1 MV are internalized by gut epithelial cells in vitro

To test whether JB-1 MV are internalized by cells in vitro, we first examined whether CFSE-labelled MV and OMV were successfully stained and retained activity by incubating them with bone marrow-derived dendritic cells (BMDCs) for 1 h. We assessed MV and OMV uptake by measuring CFSE signal in flow cytometry and found that > 85% of BMDCs were CFSE-positive for both EcN OMV and JB-1 MV (Supplementary Fig. S1), consistent with phagocytosis and suggesting that vesicles remained immunologically active after CFSE labelling.

**Figure 2:**
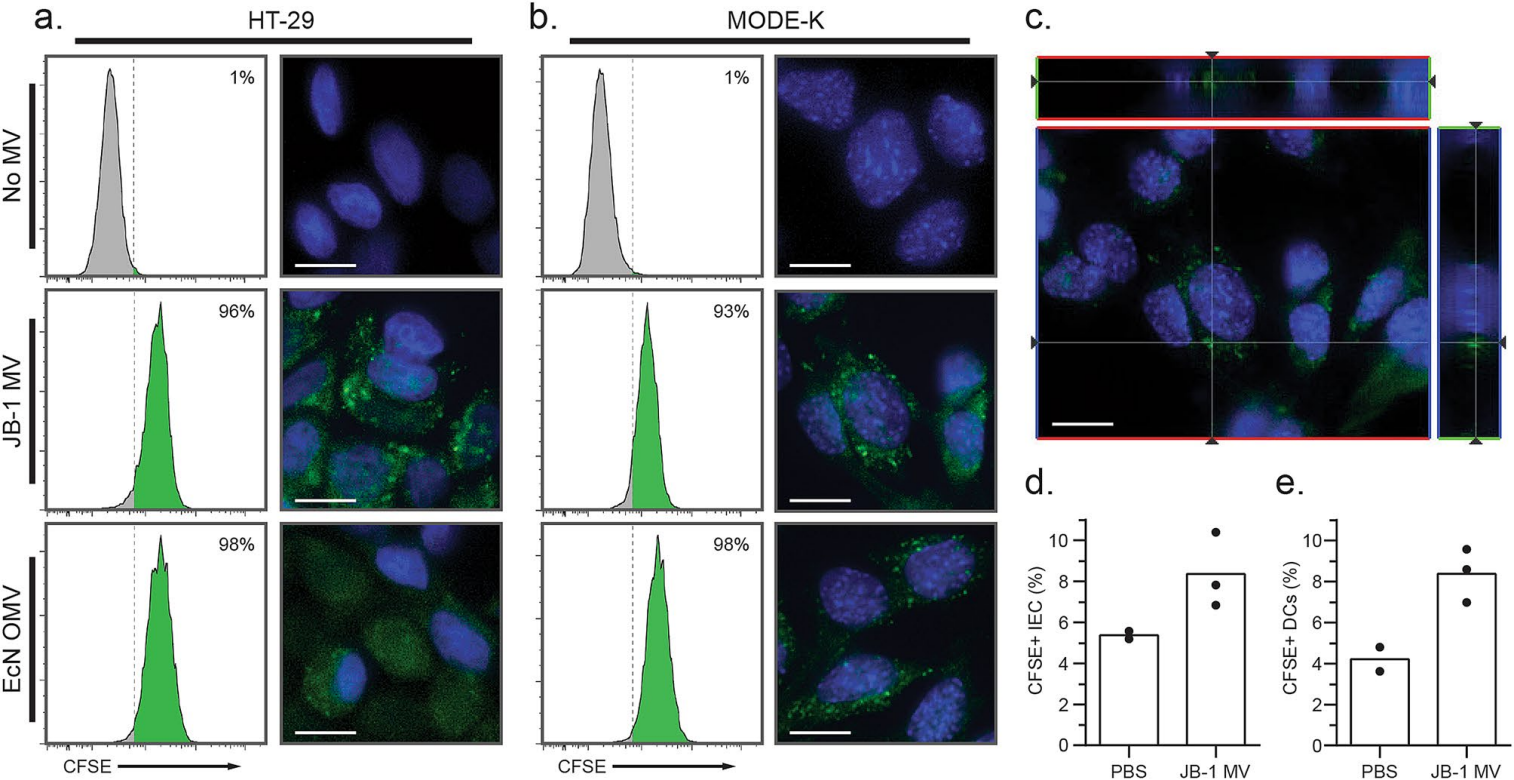
*L. rhamnosus* MV are internalized within 2 h by intestinal epithelial cells. (**a–c**) Approx. 3 × 10^10^ CFSE-labelled JB-1 MV or EcN OMV were incubated at 37 °C for 2 h with HT-29 cells (**a**) or MODE-K cells (**b**,**c**), and cell-associated fluorescence was measured by flow cytometry (left) or fluorescence microscopy (right). (**c**) Representative z-stack demonstrating CFSE fluorescence adjacent to a MODE-K nucleus after incubation with CFSE-labelled JB-1 MV. Crosshairs represent the reconstructed slices shown on the upper and right side of the image, while lines through the slices represent the z-plane shown in the main image. (**d**,**e**) Approx. 3 × 10^10^ CFSE-labelled JB-1 MV or phosphate-buffered saline (PBS) vehicle were orally gavaged to BALC/c mice, then 2 h later jejuna were isolated, and fluorescence was measured by flow cytometry of (**d**) A33^+^ CD45^-^ intestinal epithelial cells or (**e**) CD11c^+^ MHC II^+^ lamina propria dendritic cells. Scale bars represent 10 μm. Green colour represents CFSE signal, while blue represents nuclear stain (Hoechst 33342).

Since EcN OMV are known to be endocytosed by HT-29 cells (a human IEC line)^23^, we used this as a positive control in exploring whether JB-1 MV would also be internalized by IEC in vitro. We incubated HT-29 cells with CFSE-labelled JB-1 MV or EcN OMV and measured internalization by association with CFSE fluorescence. Flow cytometry analyses revealed > 96% of cells were positive for MV-related fluorescence (Fig. 2a, left panels) and clear puncta were visible within cells when viewed with fluorescence microscopy (Fig. 2a, right panels). We repeated these experiments using the mouse duodenal cell line MODE-K, and again found that both JB-1 and EcN MV were internalized to similar extents (Fig. 2b). These MV appear to be intracellular as CFSE fluorescence was consistently found near nuclei and dispersed through the cell when examined by z-stacking (Fig. 2c).

### *L. rhamnosus* JB-1 MV are internalized by gut epithelial cells in vivo

Given that MV are internalized in vitro, we wished to see if this also occurred in vivo. We gavaged BALB/c mice with CFSE-labelled JB-1 MV, then collected jejuna after 2 h. To differentiate IEC from phagocytes, we physically separated and independently isolated IEC and lamina propria cells by differential gradient separation. By flow cytometry, we identified IEC as CD45-negative (marker of differentiated hematopoietic cells) and A33-positive (intestinal epithelial cell marker^24^). We identified lamina propria DCs by positivity for CD11c and MHC II. Analyses of these populations indicated appreciable and similar fluorescence in both IEC (Fig. 2d) and lamina propria DCs (Fig. 2e), suggesting that each internalized MV. This is consistent with active internalization of JB-1 MV by IEC and DCs in vivo.

### *L. rhamnosus* JB-1 MV are likely internalized by clathrin-mediated endocytosis

OMV may be internalized by epithelial cells through a variety of mechanisms^25^. When this work was undertaken, one published study had shown that MV from the gram-positive bacterium *Staphylococcus aureus* are internalized by HeLa cells via cholesterol-dependent membrane fusion^26^. Since EcN OMV are internalized by HT-29 in a clathrin-dependent manner^23^, we tested if the same mechanism is active here and focused on JB-1 MV.

**Figure 3:**
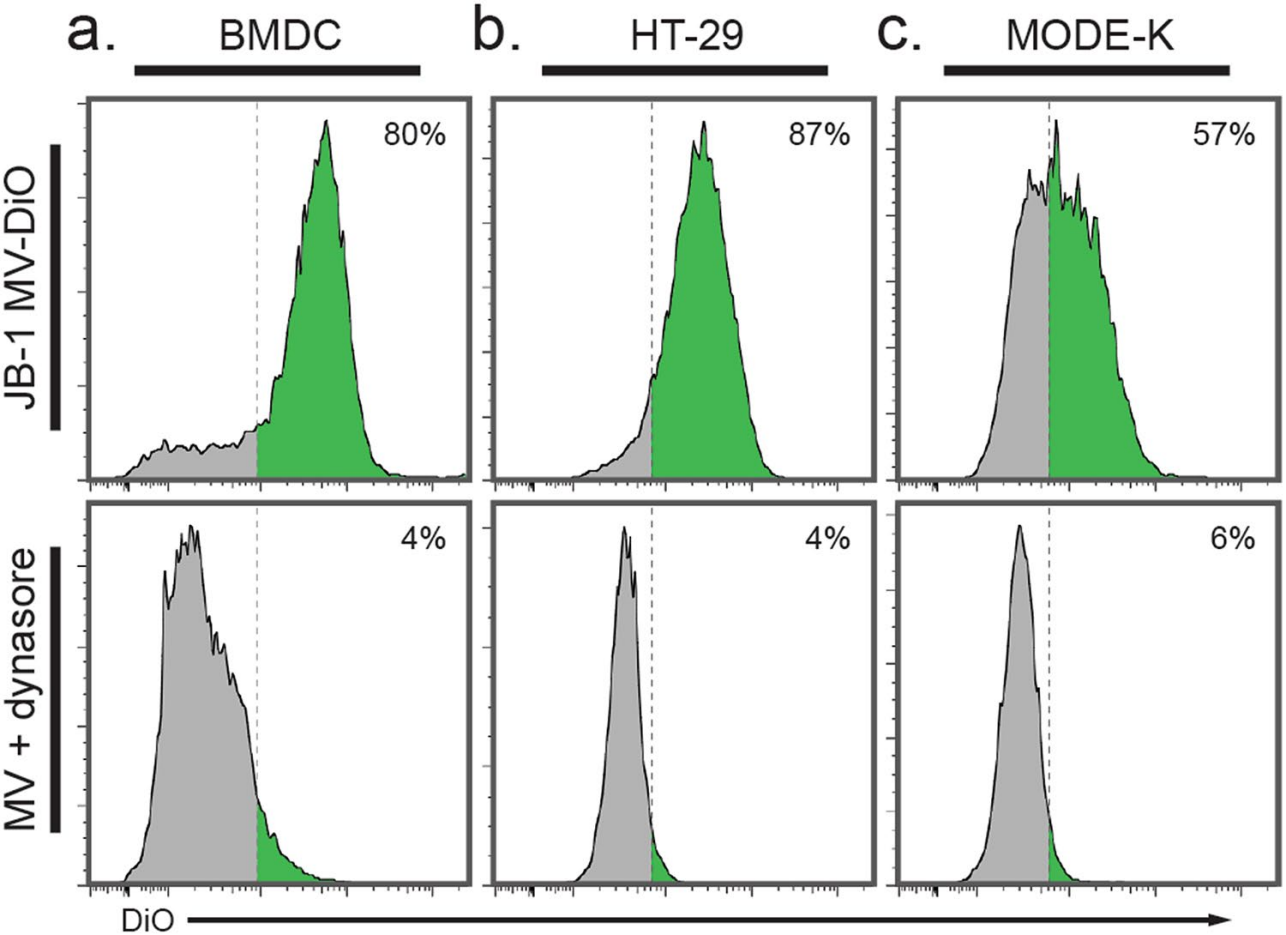
*L. rhamnosus* JB-1 are endocytosed in a likely clathrin-dependent manner. (**a–c**) Approx. 3 × 10^10^ DiO-labelled JB-1 MV were incubated with (**a**) BMDCs for 1 h or (**b**) HT-29 or (**c**) MODE-K cells for 2 h, either after preincubation with the dynamin inhibitor dynasore (bottom panels) or without preincubation (top panels).

We pre-incubated cells with the dynamin inhibitor dynasore, which blocks phagocytosis^27^ and clathrin-mediated endocytosis^28^, then added CFSE-labelled MV as before. We encountered an unexplained interaction between dynasore and CFSE causing an artifactual increased fluorescent signal that we could not prevent. We therefore labelled the MV with DiO, a lipid-soluble membrane marker previously used with dynasore in analogous experiments^29^. DiO-labelled MV were internalized by 80% of BMDCs, and this was prevented by preincubation with dynasore (Fig. 3a). Similarly, both HT-29 (Fig. 3b) and MODE-K cells (Fig. 3c) were prevented from internalization of DiO-labelled JB-1 MV by dynasore, suggesting that internalization of JB-1 MV by IEC is an active and likely clathrin-mediated process.

### *L. rhamnosus* JB-1 MV contain immunologically active lipoteichoic acid

**Figure 4:**
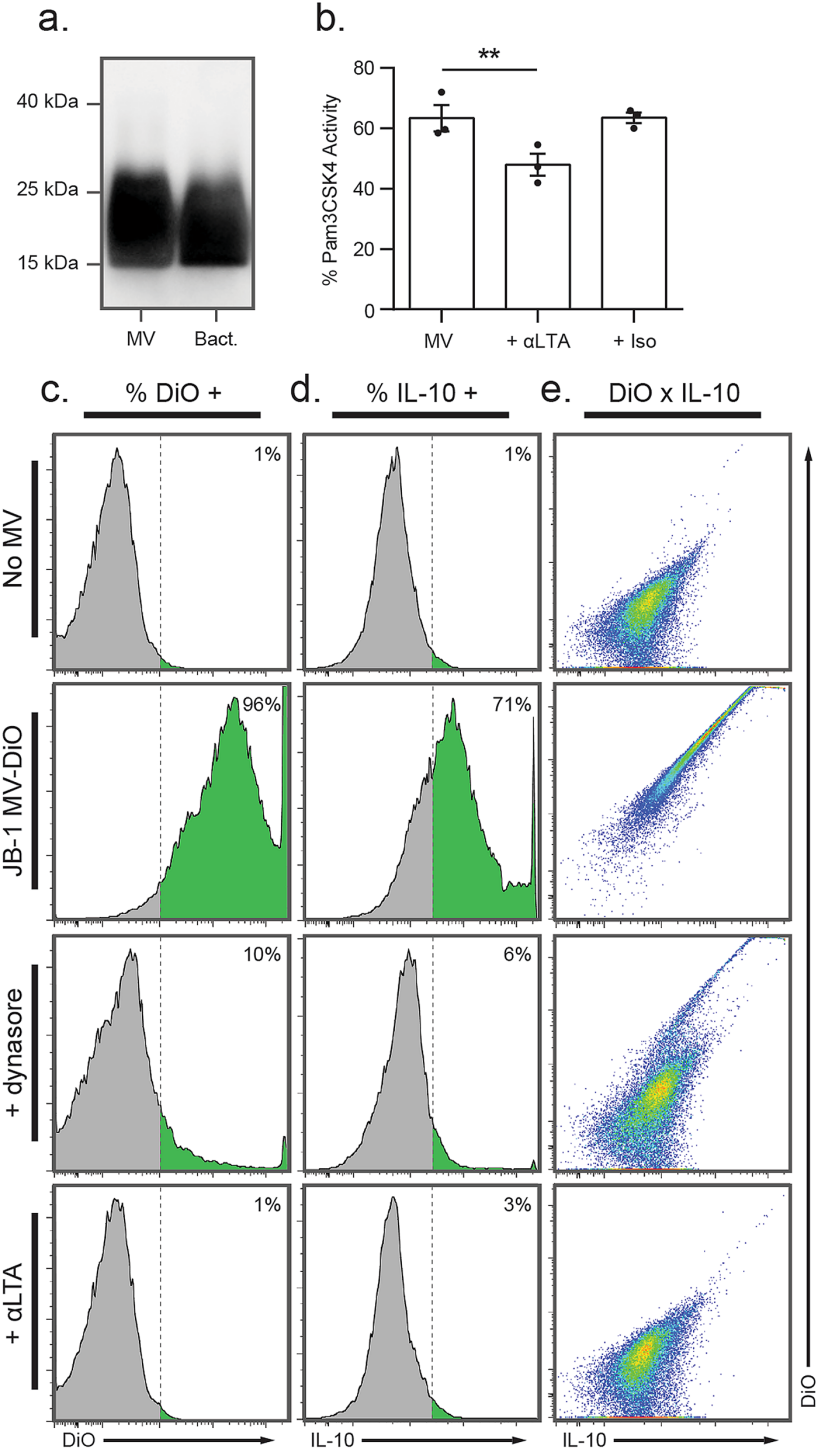
Lipoteichoic acid in *L. rhamnosus* JB-1 MV is responsible for immunomodulatory effects. (**a**) Western blot analysis was used to measure LTA associated with JB-1 MV (MV) or in lysates of whole JB-1 bacteria (Bact.). (**b**) Independent preparations of JB-1 MV were incubated with a TLR2 reporter cell line with or without preincubation with anti-LTA antibody (+ αLTA) or isotype control (+ Iso), and TLR2 activity was expressed as a percentage of that measured for the synthetic TLR2 ligand Pam3CSK4 (300 ng/mL). (**c**–**e**) DiO-labelled MV or vehicle were incubated with BMDCs for 18 h either with or without preincubation with dynasore or anti-LTA antibody, then (**c**) DiO-related or (**d**) IL-10-related fluorescence were measured by flow cytometry. (**e**) The extent to which MV internalization was associated with IL-10 expression was assessed by plotting cells as a function of DiO and IL-10 signal. Error bars represent ± 1 standard error. **p < 0.01.

Recent work suggests that MV from some lactic acid bacteria contain LTA^30^, a known ligand for TLR2 that can induce IL-10 production by DCs^31^. As we previously showed JB-1 MV to have these same effects^17^, we questioned whether LTA could mediate them and additionally serve as a ligand to induce receptor-mediated endocytosis. Using western blot, we first demonstrated the presence of LTA in JB-1 and its MV (Fig. 4a). We then performed antibody neutralization experiments to determine whether LTA is involved in MV-related effects in vitro. Anti-LTA antibodies, but not isotype control (nonspecific IgG1), inhibited MV interaction with TLR2 in a reporter cell assay (*t* = 10.1, *d* = 2.0, *p* = 0.0048; Fig. 4b). They also inhibited internalization of DiO-labelled JB-1 MV to a similar extent as dynasore (Fig. 4c), and abolished MV-induced production of IL-10 (Fig. 4d). Interestingly, colocalization measurements by flow cytometry show that IL-10 signal is correlated with internalized MV, which is largely abolished in experiments with both anti-LTA and dynasore (Fig. 4e). This suggests that internalization of whole MV is involved in the induction of IL-10.

Given that LTA mediates immune phenotypic change in DCs, we tested possible involvement of LTA in internalization by MODE-K cells but found no effect of anti-LTA antibodies (Supplementary Fig. S2). We further attempted to block the mouse pattern recognition receptors TLR2 and SIGN-R1 with neutralizing antibodies as we have done previously with JB-1 MV in DCs^17^, but again found no effect (Supplementary Fig. S2). This suggests that some other ligand-receptor systems are involved in inducing endocytosis of JB-1 MV.

## Discussion

Membrane vesicles are promising mediators of bacterial-host communication because they enable the delivery of diverse signaling molecules, including proteins, lipids, carbohydrates, and nucleic acids, to diverse recipient cells, potentially allowing for more complex signalling and protecting contents from degradation^12^. Though well-studied in multicellular eukaryotes, only recently have MV been considered as possible mediators of communication between beneficial bacteria and their host.

MV from intestinal microbes are thought to affect the local gut environment. Most work thus far has examined the role of MV in pathogenic effects of especially gram-negative bacteria^25^, while more recent work has considered the interaction between intestinal epithelial cells and MV from beneficial bacteria. Some of the first work to this end focused on the gram-negative beneficial bacterium *E. coli* Nissle 1917 (EcN), whose OMV were shown to be endocytosed by IEC in a clathrin-dependent manner^23^. While the current paper was in preparation, two recent reports found similar results in gram-positive non-pathogenic bacteria. Rubio and colleagues found that *Bacillus subtilis* MV were internalized and apparently transcytosed by Caco-2 cells in vitro^32^, while Bajic and colleagues demonstrated that MV from *Lactiplantibacillus plantarum* BGAN8 are endocytosed by HT-29 cells in a clathrin-dependent manner^33^.

Here we show that labelled MV from the gram-positive beneficial bacterium *L. rhamnosus* JB-1 are internalized by both murine and human IEC within 2 h in vitro, as evidenced both by flow cytometry experiments and by the presence of distinct puncta when viewed under routine fluorescence microscopy and z-stacking. We further showed evidence of JB-1 MV being internalized in vivo within 2 h after oral consumption of CFSE-labelled MV by both small intestinal gut epithelial cells and mononuclear dendritic cells in the lamina propria. While we have shown in a previous publication that labelled MV were internalized by cells in Peyer’s patches 18 h after feeding^17^, in the present study visible Peyer’s patches were excised prior to isolation of cells for analysis and flow cytometric analysis revealed their dendritic cell nature.

Classically, internalization of MV by IEC is thought to occur via several different mechanisms, including macropinocytosis, clathrin-dependent endocytosis, clathrin-independent endocytosis, and membrane fusion^25^. Interestingly, though rarely acknowledged, phagocytosis (e.g., of pathogens like *Salmonella typhimurium* and *Staphylococcus aureus*) can also occur in IEC^34^, though there is no evidence for this with non-pathogenic bacteria. Other investigations of the mechanism of internalization of MV from potentially beneficial bacteria have concluded that endocytosis is primarily clathrin-mediated, as internalization was inhibited by dynasore and chlorpromazine but not filipin III or nystatin^23,33^.

In microscopy experiments, we found that internalization of JB-1 MV was indistinguishable from that of EcN OMV, which suggested that similar mechanisms might be involved. Indeed, pre-incubation of both human and mouse IEC lines with the dynamin inhibitor dynasore almost entirely abolished endocytosis of JB-1 MV, suggesting that their endocytosis is clathrin-mediated. It is important to note, however, that in addition to clathrin-mediated endocytosis, dynasore also inhibits activity of dynamin required for phagocytosis^27^. Thus, we cannot distinguish between these mechanisms. We were also unable to determine which ligand-receptor systems were involved in internalization, as antibodies to LTA, TLR2, and SIGN-R1 were without inhibitory effect in MODE-K cells.

MV from several lactic acid bacteria have been shown to contain distinct cargo^33,35^, which in some cases associate with their functional effects^17^. LTA is one promising candidate, as it is present on MV of some lactic acid bacteria^30^, including the *L. rhamnosus* strain ATCC 7469^36^. Using western blot, we found that MV from *L. rhamnosus* JB-1 also contain LTA, and that it appears to be a major TLR2 agonist associated with MV as antibody neutralization experiments with anti-LTA reduced TLR2 activation in a reporter cell line. Moreover, anti-LTA inhibited internalization of JB-1 MV by BMDCs and simultaneously reduced their induction of IL-10 expression, suggesting that LTA is involved in immunoregulatory effects of JB-1 MV and that these are magnified by MV internalization.

Lipoteichoic acids are amphiphilic membrane-anchored polymers associated with the cell wall of gram-positive bacteria^37^. They are important for bacterial physiology and host–bacteria interaction, and are commonly considered analogous to the gram-negative lipopolysaccharide, as both are studied as highly immunologically active molecules in host–bacteria interactions^38^. LTA are structurally variable between species^37^, and evidence is accumulating that LTA from some bacteria are immunoregulatory^31,39–42^.

The most extensively studied receptor for LTA is TLR2, though interactions with other receptors have been documented^37^. Interestingly, structurally distinct LTAs appear to interact with TLR2 to different extents and are thought to contribute to varied response magnitudes associated with different bacteria^43^. The potential role for TLR2 signalling in MV-associated LTA is interesting considering this receptor’s known role in microbiota-host homeostasis. Round and colleagues demonstrated that colonization of the gut by *Bacteroides fragilis* requires TLR2 stimulation by polysaccharide A resulting in mucosal tolerance to the bacterium^44^. Subsequent experiments found that similar effects were seen using *B. fragilis* OMV alone, which induced IL-10 production by DC via OMV-associated polysaccharide A interacting with TLR2^14^.

JB-1 MV in vitro and in vivo have immunoregulatory activity through their effect on the generation and function of both regulatory T cells and dendritic cells^17^. We show here that this may be largely dependent on their content of LTA. Indeed, the endocytosis of JB-1 MV suggests that transcytosis may also occur in vivo, and circulating MV with LTA could thus explain some systemic effects^17–19,45^ associated with JB-1 treatment. Overall, these observations have added to our understanding and the characterization of many factors responsible for some of the immune effects of *Lacticaseibacillus rhamnosus* JB-1.

The gram-positive organism we have used in the present experiments is a useful model for exploring mechanisms of action of beneficial bacteria and possibly predicting the importance of similar molecular pathways for candidate and actual probiotics. We have underlined the importance of bacterial membrane vesicles in communication with the host. Transcytosis of epithelium in the gut and other tissue sites by bacterial MV may help explain some of the distant and systemic effects of many bacteria including probiotics.

## Methods

### Animals

Male 8- to 10-week-old specific pathogen-free BALB/c mice were purchased from Charles River (Montreal, Canada) and maintained on a 12-h light–dark cycle free access to food and water. Mice were euthanized by decapitation. All experiments involving mice were approved by the McMaster Animal Research Ethics Board and followed both the Canadian Council on Animal Care guidelines and the ARRIVE guidelines.

### Bacteria and MV preparation

*Lacticaseibacillus rhamnosus* JB-1 (JB-1) was grown from stock in 500 mL Man–Rogosa–Sharpe (MRS) medium at 37 °C in anaerobic conditions. *Escherichia coli* strain Nissle 1917 (EcN) was a gift from Ardeypharm GmbH (Herdecke, Germany) and was grown in 500 mL LB (Lennox) in aerobic conditions at 37 °C with shaking. After 24 h, cultures were centrifuged at 4 °C and 1900×*g* for 45 min to pellet bacteria. Supernatants were vacuum filtered through 0.20 μm filter units. The resulting filtrates were ultracentrifuged at 42,000 RPM (138,000×*g*) for 3 h at 4 °C in a Type 45 Ti fixed-angle rotor (Beckman Coulter, Mississauga, Canada), pellets resuspended in cold PBS, then ultracentrifuged again at 42,000 RPM (121,000×*g*) for 3 h at 4 °C in a Type 70 Ti fixed-angle rotor (Beckman Coulter). Pellets were finally resuspended in 5 μL PBS for every 1 mL of ultracentrifuged supernatant (i.e., concentrated 200x), aliquoted, and frozen at − 80 °C until further use. Protein concentrations of MV preparations were determined using the Pierce Rapid Gold bicinchoninic acid assay (Thermo Scientific, Mississauga, Canada).

To fluorescently label them, MV were incubated with 20 μm CFSE (CFDA SE; Invitrogen, Burlington, Canada) or 20 μm DiO (Invitrogen) in the dark for 20 min at 37 °C. Samples were diluted in cold PBS, ultracentrifuged to wash, then resuspended in equal volume PBS and stored at − 80 °C. To ensure the absence of nanoparticles in the original dye stocks, negative controls for all experiments were created by incubating the same concentration of CFSE or DiO with an equal volume of sterile PBS, then ultracentrifuging and resuspending as above. These negative controls did not produce fluorescence when incubated with any cell lines.

### Nanoparticle tracking analysis

MV were characterized by nanoparticle tracking analysis (NTA) using a NanoSight NS300 (Malvern Panalytical, Montreal, Canada) at the Structural & Biophysical Core Facility at the Hospital for Sick Children (Toronto, Canada). MV were diluted in PBS to 30–100 particles per frame then continuously flowed by syringe pump through a 532 nm laser. Five 60 s recordings (camera level 16) were analysed using NTA software (v. 3.2; Malvern Panalytical) with a detection threshold of 5.

### Electron microscopy

Electron microscopy was performed by the Canadian Centre for Electron Microscopy (McMaster University, Hamilton, Canada). Samples were deposited (3.5 μL) onto formvar-coated copper grids and incubated for 10 min. Excess liquid was blotted, samples were negatively stained with 1% aqueous uranyl acetate (3.5 μL) to each grid, incubated for 1 min, then blotted and dried by evaporation. Grids were viewed in a 1200 EX TEMSCAN transmission electron microscope (JEOL, Peabody, USA) operating at an accelerating voltage of 80 kV. Images were acquired with a 4-megapixel digital camera (Advanced Microscopy Techniques, Woburn, USA).

### Cell culture

All cell lines were grown in 5% CO_2_ at 37 °C, passaged at 80% confluence, and discarded after 20 passages. HT-29 (human colonic epithelial) cells and MODE-K (mouse duodenal epithelial) cells were a gift from Dr. Ali Ashkar (McMaster University). HT-29 cells were cultured in DMEM/F-12 with l-glutamine, HEPES, 100 U/mL penicillin, 100 μg/mL streptomycin, and 10% fetal bovine serum (FBS). MODE-K cells were cultured in DMEM with l-glutamine, HEPES, 100 U/mL penicillin, 100 μg/mL streptomycin, and 10% FBS. HEK-Blue mTLR2 cells were obtained from InvivoGen (San Diego, USA) and cultured in DMEM with l-glutamine, 100 U/mL penicillin, 100 μg/mL streptomycin, 100 μg/mL Normocin, and 10% FBS.

BMDCs were derived as previously described^17,46^ using tibia and femurs from BALB/c mice. Cells were plated in 100 mm dishes at 10^6^/mL in 20 mL growth medium (day 0), refreshed on days 2 and 6, and harvested on day 7.

### In vitro internalization assays

Cells were cultured to 80–90% confluence on glass coverslips or in cell culture plates (approx. 6 × 10^5^ cells/well). Where appropriate, cells were preincubated with anti-LTA (5 μg/mL; clone G43J, Invitrogen), anti-SIGN-R1 (10 μg/mL; Invitrogen), or anti-mouse TLR2 (2 μg/mL; InvivoGen) for 1 h, or dynasore (80 μM; Abcam, Toronto, Canada) for 30 min, then incubated with approx. 3 × 10^10^ CFSE-labelled JB-1 MV or EcN OMV for 2 h. Cells were then washed twice in PBS. For flow cytometry analysis cells were dissociated in Accutase for 15 min, washed once, and resuspended in 2% FBS in PBS. Cells were then analysed by flow cytometry using a FACSCelesta flow cytometer (BD Biosciences, San Jose, USA), and data analysed in FlowJo (v. 9.4; BD Biosciences).

For fluorescence microscopy, cells on coverslips were fixed for 15 min in 4% formaldehyde in PBS, washed twice, mounted with ProLong Glass antifade mountant with NucBlue nuclear stain (Hoechst 33342; Invitrogen), allowed to cure for 24 h, then imaged using a Zeiss Axio Imager Z1 microscope and processed using AxioVision software (v. 4.8; Zeiss, Toronto, Canada).

### TLR2 assay

TLR2 ligand presence was determined using a mouse TLR2 reporter cell line (HEK-Blue-mTLR2; InvivoGen) following manufacturer’s directions and as described previously^17^. Where appropriate, cells were pre-incubated with anti-LTA antibody or nonspecific isotype control (mouse IgG1; 5 μg/mL) for 1 h then incubated with 10 μL sample in 90 μL media at 37 °C for 20 h. Positive control wells were incubated with the TLR2 agonist Pam3CSK4 (300 ng/mL). Cell-free supernatants (20 μL) were then added to the detection reagent (180 μL), incubated at 37 °C for 1 h, and measured spectrophotometrically at 650 nm.

### BMDC flow cytometry analysis for IL-10 expression

BMDCs were seeded in 6-well plates at 10^6^ cell/well in 450 μL antibiotic-free RPMI. Where appropriate, cells were preincubated with anti-LTA antibody (5 μg/mL) for 1 h or dynasore (80 μM) for 30 min, then 50 μL sample were added to BMDCs and incubated at 37 °C for 18 h. Cells were suspended by cell scraper, washed, Fc blocked for 15 min, then incubated for 30 min with anti-CD11c-PerCP-Cy5.5 (1:200; Invitrogen). After washing, cells were fixed and permeabilized with BD Cytofix/Cytoperm kit (BD Biosciences) per manufacturer’s directions. Finally, BMDCs were incubated for 30 min with anti-IL-10-PE (1:200; Invitrogen) to label intracellular cytokines and analysed by flow cytometry.

### Western blot

JB-1 bacteria were lysed with 2× B-PER lysis reagent (Thermo Scientific). JB-1 lysates (approx. 600 μg/mL protein) or unlysed JB-1 MV (approx. 3 × 10^11^ MV/mL) were then heated at 95 °C for 15 min in β-mercaptoethanol-containing sample buffer. Samples were cooled and 10 μL electrophoresed in a 10% acrylamide gel, then transferred to a PVDF membrane. Blots were blocked in 5% BSA in TBS-T, then labelled with mouse IgG1 anti-LTA (G43J; Invitrogen) at 1:100. After washing, blots were then incubated with goat anti-mouse IgG1-HRP (Abcam) at 1:2000, washed again, then imaged with chemiluminescent substrate in a ChemiDoc Touch Imaging System (Bio-Rad). The full-length blot is shown in Supplementary Fig. S3.

### Measuring JB-1 MV-CFSE internalization by IEC and DCs in vivo

Mice were gavaged with approx. 3 × 10^10^ CFSE-labelled JB-1 MV in 200 μL PBS or with PBS alone, then 2 h later were euthanised and jejuna removed. Single-cell suspensions of IEC and lamina propria DC were then prepared as previously described^47,48^ and kept dark where possible to limit photobleaching. Tissues were stripped of mesentery and visible Peyer’s patches excised, flushed with cold PBS, cut into segments, suspended in 30 mL Hank’s balanced salt solution (Ca^2+^ and Mg^2+^ free) with 5% FBS, 1 mM DTT, and 5 mM EDTA, then incubated in a shaking water bath for 30 min at 37 °C to dissociate epithelial cells. Suspensions were then filtered successively through 70 μm and 40 μm cell strainers, separating dissociated IEC from lamina propria. IEC suspensions were washed twice then incubated with rabbit anti-mouse A33 polyclonal antibody (1:100; Invitrogen) for 1 h and goat anti-rabbit IgG (APC, 1:50; Invitrogen) and anti-mouse CD45-APC-Cy7 (1:100; Invitrogen) for 45 min, then analysed by flow cytometry.

To isolate lamina propria, non-dissociated jejunal segments were collected from the first 70 μm strainers and cut into smaller pieces. These were then suspended in 20 mL RPMI with 5% FBS, 1 mg/mL collagenase IV/dispase (Invitrogen), and 40 μg/mL DNAse I (Roche, Mississauga, Canada) and incubated in a shaking water bath at 37 °C for 45 min. Resultant suspensions were filtered though a 40 μm strainer, washed once with cold PBS, then applied to a Percoll (GE Healthcare, Mississauga, Canada) gradient (top layer 30%, bottom layer 75%) and centrifuged at 540×*g* for 20 min at room temperature. The cells at the interface were collected, washed twice with PBS, then antibody-labelled with anti-mouse CD11c-PerCP-Cy5.5 (1:200; Invitrogen) and MHC II-APC (1:200; Invitrogen) for 30 min. Cells were fixed and permeabilized with BD Cytofix/Cytoperm kit, intracellular cytokines labelled with anti-mouse IL-10-PE (1:200; Invitrogen) for 30 min, and finally analysed by flow cytometry.

### Data analysis

Data were analysed in R (v. 3.4.4)^49^ using the effsize package^50^. Pairwise comparisons were done by directional t-test with Welch’s correction for unequal variances (*t*) with effect size reported as Hedge’s g (*d*), with each datapoint corresponding to data from independent MV preparations. Graphs were created using the R package ggplot2^51^, GraphPad Prism (v. 6.01), or FlowJo (v. 9.4).

## Supplementary Information

Supplementary Information.

## Data Availability

Data generated during the current study are present in the supplementary section or are available from the corresponding author on request.
