# Supplementary material for: Membrane vesicles of Lacticaseibacillus rhamnosus JB-1 contain immunomodulatory lipoteichoic acid and are endocytosed by intestinal epithelial cells
Source: Sci Rep. 2021 Jul 2;11:13756. doi: 10.1038/s41598-021-93311-8 (PMC8253831; doi:10.1038/s41598-021-93311-8)
Supplement: Supplementary file 1 — Supplementary Information. [file 41598_2021_93311_MOESM1_ESM.pdf]

Supplementary Information:

Membrane vesicles of *Lacticaseibacillus rhamnosus* JB-1 contain immunomodulatory lipoteichoic acid and are endocytosed by intestinal epithelial cells

Kevin Champagne-Jorgensen [1,2]*, M. Firoz Mian [2], Karen-Anne McVey Neufeld [2,3], Andrew M. Stanisz [2], John Bienenstock [2,3]

[1] Neuroscience Graduate Program, McMaster University, Hamilton, ON, Canada
[2] Brain-Body Institute, St. Joseph's Healthcare Hamilton, Hamilton, ON, Canada
[3] Department of Pathology and Molecular Medicine, McMaster University, Hamilton, ON, Canada

*Corresponding author: champk1@mcmaster.ca

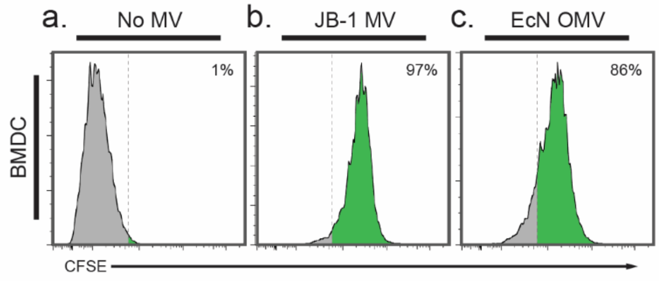

Supplementary Figure S1. ***L. rhamnosus*** **JB-1 MV are internalized within 1 hour by bone marrow-derived dendritic cells.** (**a**) Either PBS vehicle or approx. $3x10^{10}$ (**b**) CFSE-labelled JB-1 MV or (**c**) EcN OMV were incubated at 37°C for 1 h with BMDCs and cell-associated fluorescence was measured by flow cytometry.

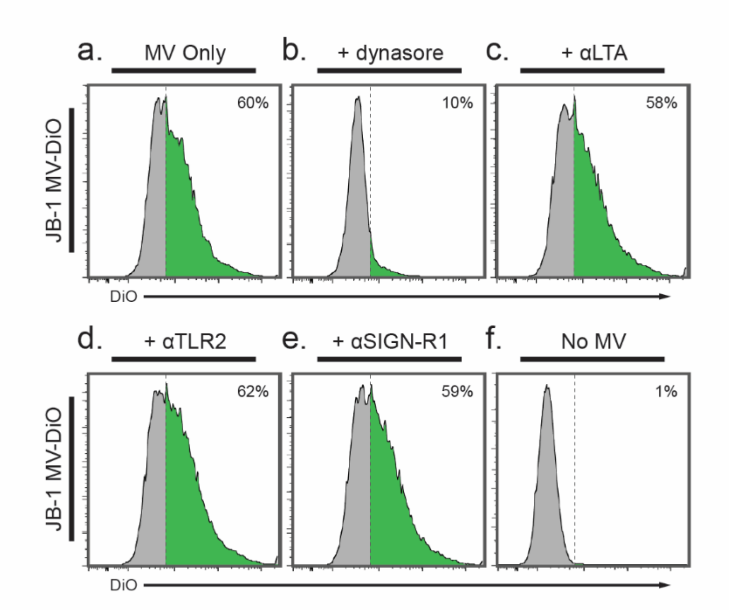

Supplementary Figure S2. *L. rhamnosus* JB-1 MV are endocytosed independent of LTA, TLR2, and SIGN-R1. (**a**-**e**) Approx. $3x10^{10}$ DiO-labelled JB-1 MV were incubated with MODE-K cells for 2 h after preincubation with (**a**) nothing added, (**b**) dynasore, (**c**) anti-LTA antibody, (**d**) anti-mouse TLR2 antibody, or (**e**) anti-SIGN-R1 antibody. (**f**) Cells were incubated only with PBS vehicle.

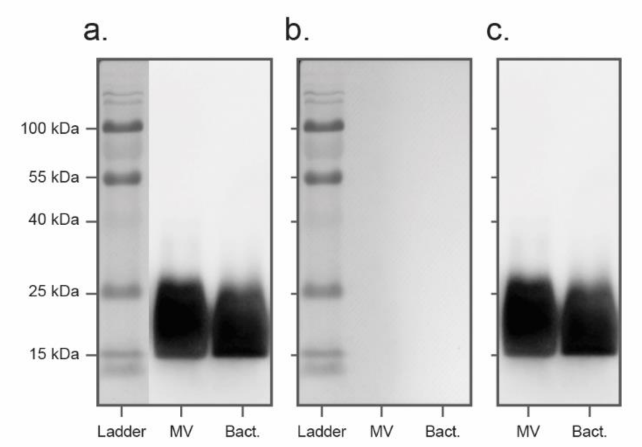

Supplementary Figure S3. *L. rhamnosus* JB-1 MV contain lipoteichoic acid. (**a**-**c**) Full-length western blot used to measure LTA associated with JB-1 MV (MV) or in lysates of whole JB-1 bacteria (Bact.). Images show (**a**) colorimetric ladder merged with chemiluminescent LTA signal, (**b**) colorimetric blot alone, or (**c**) chemiluminescent signal from sample-containing wells.
